# Cost Impact of a Coronary Computed Tomography Angiography-Based Diagnostic Pathway for Low-Risk Chest Pain in the Emergency Department

**DOI:** 10.31083/RCM50184

**Published:** 2026-07-21

**Authors:** Ryan Karlsson, Michael Cronin, Niall Sheehy, Geraldine McMahon, Ross T Murphy, Caroline Daly

**Affiliations:** ^1^Department of Cardiology, St James’s Hospital, D08 NHY1 Dublin, Ireland; ^2^Department of Radiology, St James’s Hospital, D08 NHY1 Dublin, Ireland; ^3^Department of Emergency Medicine, St James’s Hospital, D08 NHY1 Dublin, Ireland

**Keywords:** coronary computed tomography angiography, emergency department chest pain, diagnostic pathway, coronary artery disease, non-invasive cardiac imaging, cost analysis

## Abstract

**Background:**

Emergency chest pain presentations represent a substantial burden on healthcare resources. The use of coronary computed tomography angiography (CCTA) has been shown to be safe and associated with reduced costs in stable outpatients referred for invasive coronary angiography (ICA). The history, electrocardiogram, age, risk factors, troponin (HEART) risk assessment score is used to risk-stratify patients with undifferentiated chest pain. We sought to evaluate the cost impact of an accelerated outpatient CCTA-based diagnostic pathway compared with inpatient ICA for the investigation of patients presenting acutely to the emergency department (ED) with symptoms suggestive of coronary artery disease (CAD) at a tertiary referral centre.

**Methods:**

We performed a comparative cost analysis based on retrospective observational data including stable adult ED patients who presented in 2023.

**Results:**

369 patients underwent outpatient CCTA and 376 were admitted for ICA. The median time to outpatient CCTA was 14 days (interquartile range (IQR) 5–24). Use of the CCTA-based pathway was associated with a 78% reduction in diagnostic pathway costs (€2990 per patient), when compared to admission for ICA, for the investigation of lower-risk individuals, with no increase in 30-day mortality or myocardial infarction. Patients referred for outpatient CCTA had a significantly shorter median length-of-stay compared with those admitted for ICA (6.5 hours (IQR 5.0–9.0) vs. 5 days (IQR 3.0–14.9); *p* < 0.05).

**Conclusions:**

Use of an accelerated outpatient CCTA-based diagnostic pathway for low-risk chest pain patients in the ED was associated with substantial cost savings and reduced hospital utilization without evidence of compromised short-term safety.

## 1. Introduction

Coronary computed tomography angiography (CCTA) has emerged as a reliable non-invasive method in the assessment of patients with stable chest pain given its high sensitivity and specificity for the detection of coronary artery disease (CAD) [1,2] with a proven diagnostic performance when compared with invasive coronary angiography (ICA) [3,4]. This is reflected by a class one recommendation by the European Society of Cardiology (ESC) in 2024 for use of CCTA as the preferred diagnostic modality to investigate individuals with suspected chronic coronary syndrome and low or moderate pre-test likelihood of obstructive CAD [5]. Use of a CCTA-based diagnostic strategy in this patient cohort has shown benefit over other non-invasive strategies in randomised trials, for example exercise electrocardiogram (ECG), with regards to rates of angina and adverse events during follow-up [6,7].

The use of CCTA in the emergency department (ED) setting has been shown to be both safe and associated with reduced resource utilization and healthcare costs [8,9]. An accelerated outpatient diagnostic CCTA pathway for chest pain presentations was developed at our centre in 2021 through collaboration between the cardiology, radiology and emergency departments with the aim of optimizing discharge time and stratifying patient risk for future cardiac events. The ED chest pain pathway which has been previously described [10], utilizes the history, electrocardiogram, age, risk factors, troponin (HEART) risk assessment score [11] to identify patients suitable for outpatient work-up, whereby low risk patients (HEART score 0–3) are either discharged or commence on the imaging pathway and high risk patients (HEART score >3) are referred for further evaluation by the cardiology team. A virtual chest pain clinic, led by advanced nurse practitioners, follows up on the results of each outpatient CCTA, and so downstream invasive angiography or revascularization can be arranged promptly as required. Our centre has previously described the outcomes of this CCTA pathway, reporting that at a median of 14 days to CCTA for low-risk chest pain presentations to the ED, the result was no mortality or myocardial infarction at 30 days, with a low level of overall coronary revascularization (2.4%) in these patients [10].

The use of CCTA as part of an outpatient diagnostic pathway for chest pain presentations represents not only a safe protocol but one potentially associated with meaningful reductions in healthcare costs. In the Cardiac CT Versus Exercise Testing in Suspected Coronary Artery Disease (CRESCENT) trial, a CCTA-guided approach yielded cost savings of 16% compared to exercise ECG testing in outpatients at one-year follow-up due to lower rates of follow-up non-invasive diagnostic testing [7]. Cost analyses of international studies such as the CONSERVE [12] and CAT-CAD [13] trials have reported significant savings through the use of CCTA in patients initially referred for ICA for the evaluation of suspected stable ischaemic heart disease, without compromising patient safety.

Given the favourable safety profile already demonstrated [10], where no major adverse cardiac events (MACE) were demonstrated at 30 days for patients on the CCTA pathway, our primary aim for this study was to describe the cost impact at our centre of an accelerated outpatient CCTA-based diagnostic pathway compared with the pathway of patient admission for ICA for the investigation of low-risk chest pain presentations to the ED.

## 2. Materials and Methods

A cost analysis was performed based on retrospective observational data at St James’s Hospital including ambulatory patients over the age of 18 who presented unscheduled to the ED with symptoms suggestive of CAD referred to the early outpatient CCTA pathway (HEART score 0–3) or admitted for invasive angiography (HEART score >3) between January 1st and December 12th 2023. Haemodynamically unstable patients, patients with ST elevation myocardial infarction, and patients transferred as inpatients from referring hospitals were excluded. Data collection was performed by junior doctors on the clinical cardiology service under the clinical governance of the department lead, using the electronic patient record including patient characteristics, comorbidities, vital signs, high-sensitivity cardiac troponin level (hs-cTn), length-of-stay (LOS), and rate of coronary revascularization. Vascular disease was defined as a history of cerebrovascular disease, peripheral arterial disease (defined as history of peripheral arterial revascularization), or ischaemic heart disease including those who had previously undergone percutaneous coronary intervention or coronary artery bypass graft surgery. 30-day events were adjudicated by the research team and escalated to department head if clinical indecision remained. All patients were followed up at 30 days, and if there was missing data in the patient file relevant to the patient’s history, the patient was contacted by the research team to specifically collect this data. As a result, a complete dataset was obtained. Data were collected on a hospital-based password-protected computer. Patients’ details were pseudonymed as part of data storage.

Average cost estimates for the various services involved in patient care were provided by the hospital’s finance department based on latest annual expenditure analysis and Health Service Executive (HSE) activity-based funding figures. Cost of outpatient cardiac magnetic resonance (CMR) stress perfusion imaging was also estimated to inform discussion regarding the potential for further expansion of non-invasive testing services. Per-patient pathway costs were estimated by summing unit costs for each component of care; for inpatient admissions, bed-day costs were calculated as median length of stay multiplied by the cardiology ward nightly rate, and downstream ICA after outpatient CCTA was incorporated probabilistically using the observed proportion proceeding to ICA. Revascularization costs were excluded to focus specifically on diagnostic pathway costs rather than downstream therapeutic interventions.

Statistical analyses were performed using IBM SPSS (Version 29.0.2.0, IBM Corporation, Armonk, NY, USA). Continuous variables were presented as medians with interquartile ranges and were compared between groups using the Mann–Whitney U test. Categorical variables were reported as counts with percentages and were compared using the χ^2^ test or Fisher exact test, as appropriate, when expected cell counts were <5. All statistical tests were two-sided, and a *p* value < 0.05 was considered statistically significant.

## 3. Results

745 patients were included in the study. 369 patients underwent outpatient CCTA and 376 patients were admitted for ICA. All patients were followed up at 30 days. The characteristics of each patient population are outlined in Table 1. The median LOS in the ED for patients referred to the outpatient CCTA pathway was 6.5 hours (interquartile range (IQR) 5.0–9.0 hours) and the median wait time to outpatient CCTA was 14 days (IQR 5–24 days). 0 patients who underwent outpatient CCTA suffered mortality or myocardial infarction at 30 days. 11% (39) of patients who underwent outpatient CCTA were referred for subsequent ICA with an overall coronary revascularization rate of 2% observed in this group.

**Table 1.** **Characteristics of CCTA population and ICA population**.

Characteristic	CCTA (n = 369)	ICA (n = 376)	*p* value
Age, years, median (IQR)	52 (44–59.5)	65 (59–73)	<0.001
Gender, n (%)	Male = 198 (53.70) Female = 171 (46.30)	Male = 263 (69.95) Female = 113 (30.05)	<0.001
Family history of IHD, n (%)	206 (56.94)	121 (32.30)	<0.001
Hypertension, n (%)	122 (33.00)	228 (60.80)	<0.001
T2DM, n (%)	34 (9.20)	87 (23.14)	<0.001
T1DM, n (%)	2 (0.50)	3 (0.80)	>0.99
Smoker, n (%)	Current = 96 (26.00) Ex = 92 (24.90)	Current = 90 (24.00) Ex = 150 (40.00)	0.512 <0.001
Dyslipidaemia, n (%)	163 (44.20)	230 (61.30)	<0.001
Vascular disease, n (%)	10 (2.70)	158 (42.10)	<0.001
Systolic BP, median (IQR)	133 (120–150)	137 (121–154)	0.195
Diastolic BP, median (IQR)	80 (76–88)	78 (72–87)	0.003
Heart rate, median (IQR)	76 (68–87)	78 (68–90)	0.075
Electrocardiogram, n (%)	Normal = 323 (87.50)	Normal = 253 (67.50)	<0.001
Troponin, n (%) or median (IQR)	Normal = 357 (96.70)	20 (1–62)	<0.001
Creatinine, median (IQR)	72 (61–85)	81 (68–99)	<0.001
Revascularization, n (%)	9 (2.40) PCI = 6 CABG = 3	164 (43.62) PCI = 136 CABG = 28	<0.001
pLAD/LMCA involvement, n (%)	5 (1.40)	85 (22.70)	<0.001
MI at 30 days, n (%)	0 (0)	0 (0)	
Death at 30 days, n (%)	0 (0)	9 (2.40)	0.004

Continuous results are expressed as a median (with interquartile range), and categorical variables are expressed as a total (with percentage). IHD, ischaemic heart disease; T2DM, Type-2 diabetes mellitus; T1DM, Type-1 diabetes mellitus; BP, blood pressure; pLAD, proximal left anterior descending artery; LMCA, left main coronary artery; MI, myocardial infarction; CCTA, coronary computed tomography angiography; ICA, invasive coronary angiography; IQR, interquartile range; PCI, percutaneous coronary intervention; CABG, coronary artery bypass graft.

Patients admitted for ICA had a median LOS of 5.0 days (IQR 3.0–14.9 days), which was significantly longer than the outpatient CCTA cohort (*p* < 0.001), and included a median wait time from hospital admission to ICA of 47.0 hours (IQR 23.8–101.0 hours). 44% (164) of patients admitted for ICA underwent revascularization. 35% (133) of patients admitted for ICA had a normal hs-cTn level of <14 ng/L. 23% (88) of patients admitted for ICA had both a hs-cTn <14 ng/L and did not undergo revascularization during ICA.

**Fig. 1.**
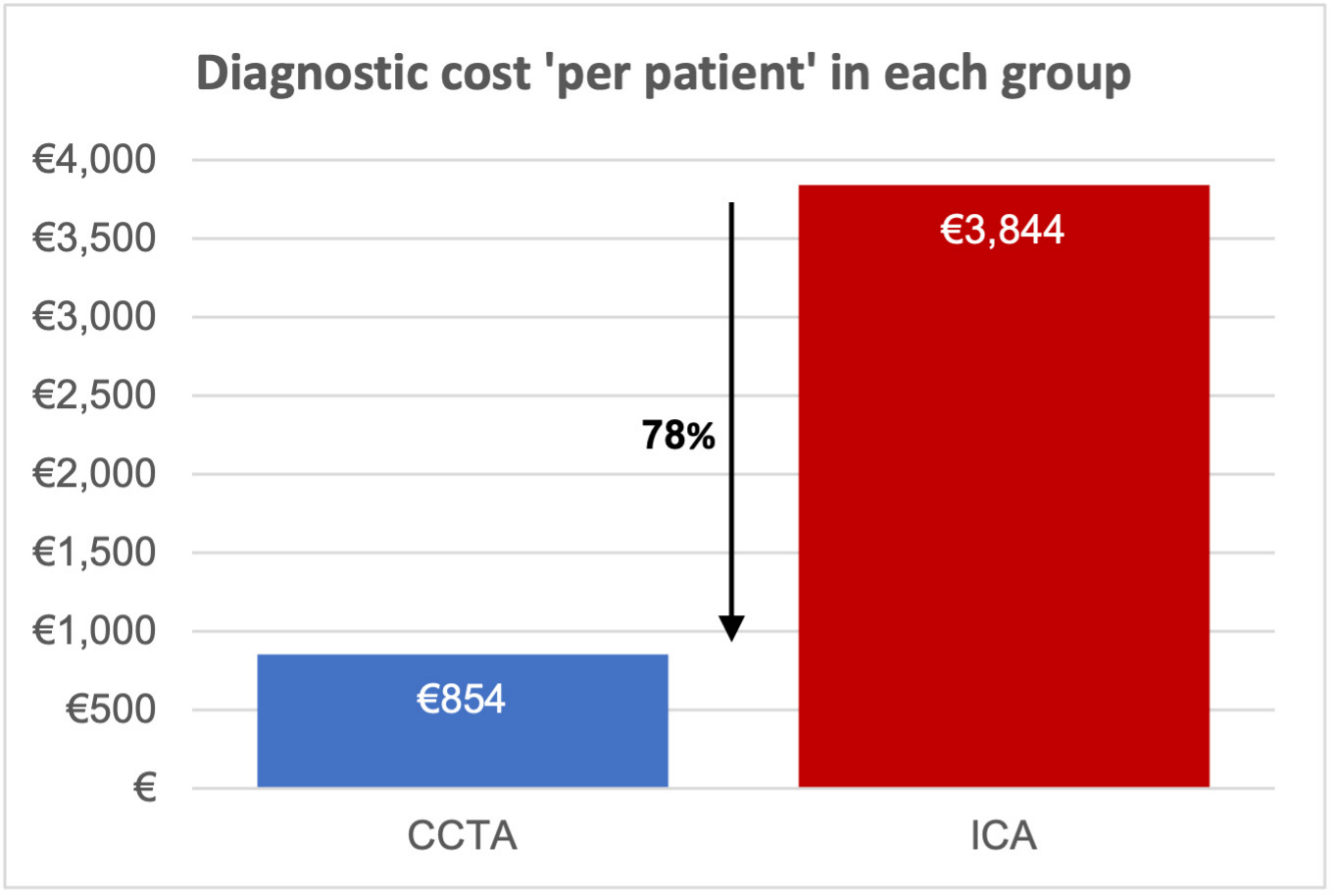
**Average diagnostic cost per patient for those investigated via the outpatient CCTA pathway (n = 369, blue) and via admission for ICA (n = 376, red)**.

The average cost for various services involved in patient care is detailed in Table 2. The estimated average cost of patient work-up via the outpatient CCTA pathway was €854 per patient, accounting for the need for subsequent ICA in 11% of patients. At a median LOS of 5 days, the estimated average cost of patient work-up via hospital admission for ICA was €3844 per patient. This represents an estimated reduction in diagnostic pathway costs of 78% or €2990 per patient through use of the CCTA pathway for the investigation of lower-risk individuals. These estimates represent diagnostic costs and so are not inclusive of the additional costs associated with proceeding to revascularization where indicated (2% of the CCTA population and 44% of the ICA population). Difference in cost is illustrated in Fig. 1.

**Table 2.** **Average cost of various services in St James’s Hospital in 2023**.

Service	Average cost per patient (€)
Emergency department attendance	521
Outpatient coronary CT angiography	210
Diagnostic invasive coronary angiography	1168
Inpatient bed on cardiology ward (per night)	431
Outpatient CMR stress perfusion imaging	420

CT, computed tomography; CMR, cardiac magnetic resonance. The average Euro to US Dollar exchange rate at that time was 1 EUR = 1.08 USD.

**Fig. 2.**
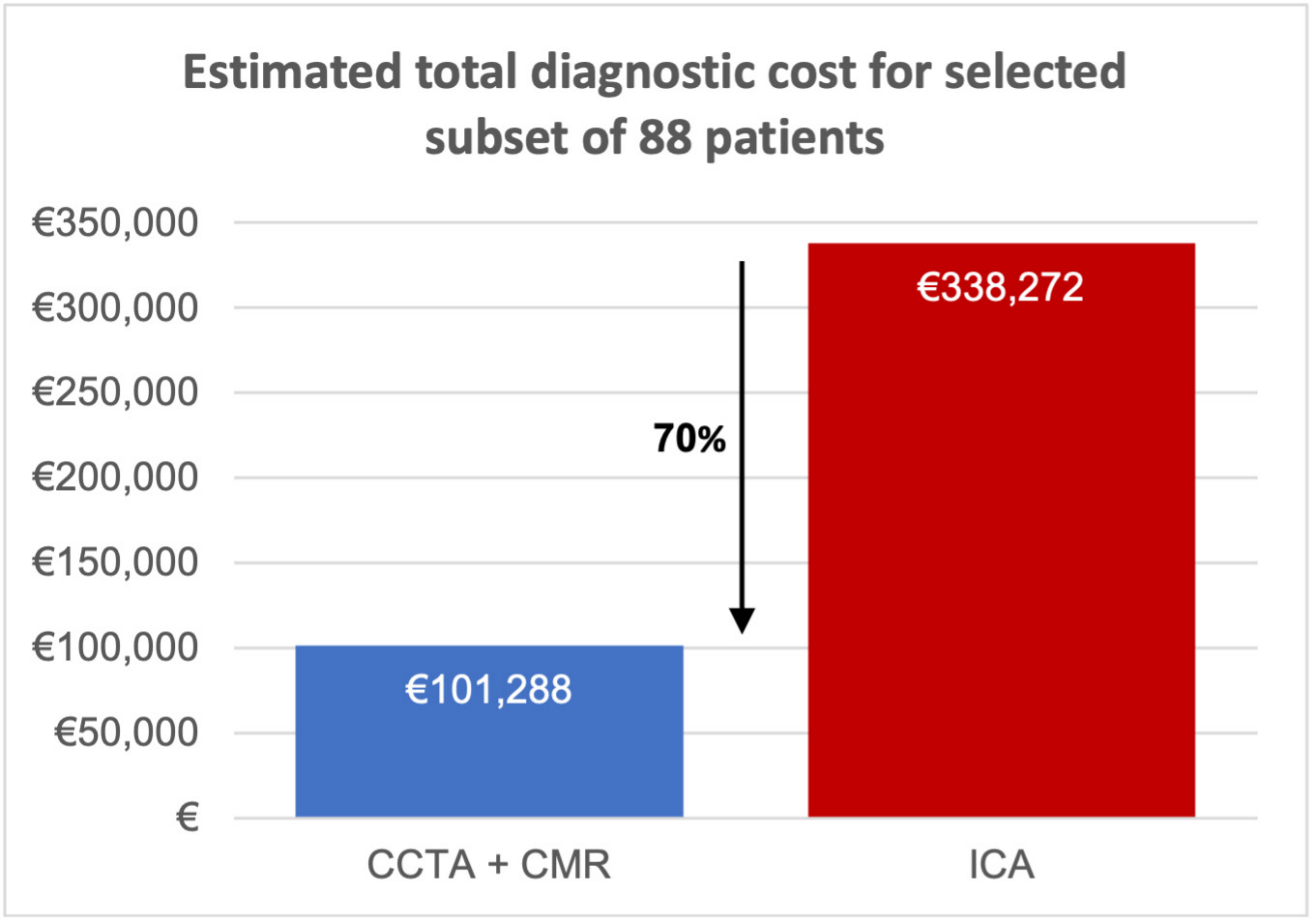
**Comparison of diagnostic cost**. Projected reduction in total diagnostic cost if outpatient comprehensive non-invasive testing (CCTA + CMR stress perfusion imaging, blue) had been utilised rather than ICA (red) for 88 patients admitted with negative serum troponin levels who did not require revascularization. The average Euro to US Dollar exchange rate at that time was 1 EUR = 1.08 USD.

88 patients who were admitted for ICA had both a negative troponin and did not require revascularization during ICA, at an estimated total cost of €338,272. Had these 88 patients instead been worked up in an outpatient setting with urgent CCTA, the estimated total cost would have been €64,328. If all 88 patients had also undergone non-invasive functional testing in the form of CMR stress perfusion imaging in addition to CCTA, the estimated total cost of comprehensive outpatient work-up for this group could instead have been €101,288, representing a potential 70% absolute reduction in diagnostic costs. While non-invasive functional testing may not be indicated in every one of these patients following CCTA, this conservative approach represents an estimated absolute saving of €236,984 per annum compared with the pathway of patient admission for invasive testing. This comparison is illustrated in Fig. 2.

## 4. Discussion

Emergency chest pain presentations represent a significant cost burden to the health service. Our study provides insight into the costings of an accelerated outpatient diagnostic pathway involving use of CCTA at a tertiary referral centre. Based on our cost analysis, the use of an outpatient ED CCTA pathway for the investigation of suspected CAD in lower-risk individuals rather than inpatient admission for ICA in 2023 resulted in a total estimated saving of €1,103,145, by means of a 78% per patient reduction in diagnostic costs. These findings are consistent with international randomised trials such as the CONSERVE trial [12], which found that 77% of patients with suspected stable ischaemic heart disease initially referred to ICA avoided invasive evaluation when undergoing CCTA with no difference in clinical outcomes and a reduction in diagnostic evaluation costs by 57%. Likewise, cost analysis of the CAT-CAD trial [13] found that the application of CCTA as a first-line diagnostic test in stable patients referred for ICA resulted in a 63% cost reduction, likely attributable to reduced numbers of invasive tests and hospitalizations.

The economic implications of CCTA-based pathways in emergency department chest pain evaluation have been explored previously, with mixed findings. Early randomized studies such as ROMICAT-II did not demonstrate a net cost benefit associated with CCTA [14], likely reflecting higher downstream testing rates and differences in patient selection and healthcare system organization at the time of study conduct. More contemporary evidence, including the BEACON trial, has demonstrated that a CCTA-based strategy is safe and associated with lower overall costs compared with standard care [15]. Our findings are consistent with these later data and extend the existing literature by providing a real-world assessment of the diagnostic cost impact of an accelerated outpatient ED CCTA pathway within the Irish public healthcare system. To our knowledge, this is the first study to quantify the potential cost savings and acute bed-day reduction associated with this model of care in Ireland. This is particularly relevant given the local pressures on inpatient capacity, delayed access to non-invasive cardiac testing, and variation in referral patterns for invasive coronary angiography.

Increasing demand for inpatient hospital beds in Ireland has placed significant pressure on the health service [16]. Use of a CCTA-based outpatient diagnostic pathway also appears to be effective in alleviating some of this burden. Lower-risk patients investigated using outpatient CCTA spent a median of just 6.5 hours in hospital and did not require an acute inpatient bed. For those admitted from the ED for invasive angiography, the hospital LOS represents a large contributor to the overall cost of diagnostic work. We found that the median LOS for this cohort was 5 days inclusive of a median wait time to ICA of 47 hours. Specifically, providing a pathway that diverts low-risk chest pain presentations from the emergency room to an outpatient pathway allowed for the sparing of an estimated 1845 acute ‘bed-days’ in 2023, with associated reductions in healthcare costs, given that the standard of care in the facility prior was to admit almost all of these patients for invasive coronary angiography.

Aside from CCTA, non-invasive functional testing is increasingly being applied worldwide for risk stratification of patients with suspected or known CAD [17]. CMR stress perfusion imaging has shown both diagnostic accuracy in detecting flow-limiting coronary lesions as well as prognostic value and has merit as a gatekeeper for ICA and percutaneous coronary intervention [17,18,19], with endorsement by the latest ESC guidelines [5]. Our analysis also highlights the potential for further diagnostic cost reduction (of up to 70%) through the use of outpatient non-invasive functional testing (namely CCTA and CMR stress perfusion imaging) over admission for ICA in a selected subset of patients.

Non-invasive cardiac testing in Ireland is an underdeveloped resource, its use limited by significant wait times within the public health service, often resulting in physician decision to instead pursue ICA with invasive functional assessment at significant financial cost. The selection of suitable patients in whom outpatient testing is warranted poses a challenge but one which holds great potential value. The findings of our study provoke thought that perhaps larger investment in early access pathways promoting outpatient initial non-invasive testing, such as CCTA, and including functional testing, such as CMR stress perfusion imaging, may not only aid in the struggle with inpatient hospital bed scarcity but also result in significant savings for the health service.

Barriers to the implementation of such a pathway might include CT scan availability, a healthcare workforce’s capacity, and pattern of referral for imaging. In our experience, a collaborative approach between the clinical cardiology, emergency department, and radiology resulting in a standard operating procedure is essential prior to the rollout of a pathway. Understanding the priorities of different clinical departments helps to create a mutually beneficial situation. The standard operating procedure should have a predesignated capacity for CT imaging defined (for example, one outpatient slot per day in our centre), with appropriately trained staff whose role is to identify suitable patients (for example, ED doctors, independent nurse practitioners, and the clinical cardiology team), as well as those who have appropriate training to perform cardiac CT imaging safely (i.e., radiographers and radiologists).

## 5. Limitations

The benefits described regarding both diagnostic cost reduction and sparing of valuable acute inpatient beds may be somewhat over-estimated in our study, given the possibility that some patients selected for CCTA may not necessarily have been admitted for ICA prior to the pathway’s development, and given the use of cost estimates which may not be totally inclusive of factors such as indirect labour. Costs in our study were calculated using standard hospital reimbursements within the public healthcare system, rather than actual itemized expenditure. Whilst this reflects real-world billing practice, it may not fully capture the true resource use of direct and indirect costs. Further, conditions such as frailty, cancer, heart failure and chronic kidney disease were not accounted for within the inclusion and exclusion criteria, which are conditions associated with a longer hospital stay, and which may affect savings observed in the analysis. Finally, observed differences in short-term mortality between patient cohorts should be interpreted as safety observations rather than comparative effectiveness outcomes, given baseline differences in clinical risk between groups. Despite these potential limitations, the economic advantages of an accelerated outpatient diagnostic CCTA pathway remain apparent.

## 6. Conclusions

Our cost analysis demonstrated substantial reductions in diagnostic pathway costs associated with a CCTA-based diagnostic pathway for the investigation of low-risk patients presenting unscheduled to the emergency department with symptoms suggestive of coronary artery disease, without evidence of compromised safety. These findings may inform expansion of outpatient non-invasive testing services or the development of similar pathways in other jurisdictions with comparable cardiovascular risk profiles.

## Data Availability

All data reported in this paper will be shared by the lead contact upon request.

## Abbreviations

CCTA, coronary computed tomography angiography; CAD, coronary artery disease; ICA, invasive coronary angiography; ESC, European Society of Cardiology; ECG, electrocardiogram; ED, emergency department; HEART, History, Electrocardiogram, Age, Risk factors, Troponin levels; LOS, length-of-stay; hs-cTn, high-sensitivity cardiac troponin level; HSE, Health Service Executive; CMR, cardiac magnetic resonance.
